# Infographic: study design of the BALATON and COMINO phase 3 randomised trials of faricimab in patients with retinal vein occlusion

**DOI:** 10.1038/s41433-024-02936-2

**Published:** 2024-02-23

**Authors:** Andrew Chang, Aachal Kotecha, Faiz Kermani, Insaf Saffar

**Affiliations:** 1 Sydney Retina Clinic, Sydney, Australia; 2 Roche Products Ltd., Welwyn Garden City, UK; 3 F. Hoffmann-La Roche Ltd., Basel, Switzerland

**Keywords:** Retinal diseases, Therapeutics

**Fig. 1:**
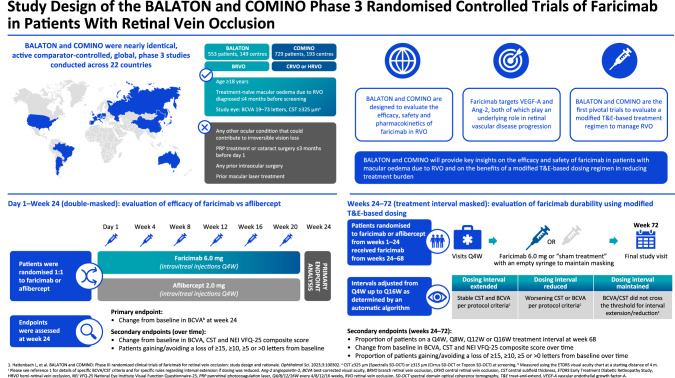
This infographic summarises the design of two nearly identical phase 3 trials of faricimab, a dual angiopoietin-2/vascular endothelial growth factor-A inhibitor, in patients with macular edema due to retinal vein occlusion: BALATON (NCT04740905) and COMINO (NCT04740931). Patients were randomised to receive faricimab 6.0 mg or aflibercept 2.0 mg monthly from day 1 to week 24 to compare the efficacy of faricimab versus aflibercept. The primary endpoint at week 24 was the mean change from baseline in best-corrected visual acuity. From weeks 24 to 72, all patients received faricimab 6.0 mg according to a modified treat-and-extend based regimen, with dosing up to every 16-weeks to evaluate treatment durability.

## References

[CR1] Hattenbach L, et al. BALATON and COMINO: Phase III randomized clinical trials of faricimab for retinal vein occlusion: Study design and rationale. Ophthalmol Sci. 2023;3:100302. 37810589 10.1016/j.xops.2023.100302 PMC10556281

